# Opinions of the Ancients on the Causes and Treatment of Cholera

**Published:** 1831-11

**Authors:** Francis Adams

**Affiliations:** Surgeon, Banchory Ternan.


THE LONDON

### Medical and Physical Journal.

393, VOL. LXVI.] NOVEMBER 1831. [65, *New Series*.

"For many fortunate discoveries in medicine, and for the detection of numerous errors, the world is indebted to the rapid circulation of Monthly Journals; and there never existed any work to which the Faculty, in Europe and America, were under deeper obligations than to the 'Medical and Physical Journal of London,' now forming a long but invaluable series."—RUSH.

### ORIGINAL PAPERS, AND CASES,

### OBTAINED FROM PUBLIC INSTITUTIONS AND OTHER AUTHENTIC SOURCES.

### CHOLERA.

*Opinions of the Ancients on the Causes and Treatment of Cholera.* By FRANCIS ADAMS, Esq., Surgeon, Banchory Ternan.

THE dreadful destruction of human life occasioned of late years, throughout Asia, by the prevalence of cholera, and the alarm excited by its appearance in the east of Europe, have conferred upon all investigations into its nature a degree of interest and importance, such as is possessed by no other subject of medical research at the present time: hence the profession has been laudably active in collecting from every source whatever information can be obtained of the various phenomena which it is capable of assuming, and of the modes of treatment which are said to have been found most effectual in warding off or alleviating so frightful an evil. Believing that the opinions of the Greek, Roman, and Arabian physicians have not obtained, in this instance, that consideration which they deserve, I have hoped to contribute something to the general fund of knowledge, by giving as comprehensive an exposition of them as the limits to which I am necessarily restricted will permit, whereby we shall be enabled to avail ourselves of the accumulated experience of many distant ages on this subject.

Hoc opus, hoc studium parvi properemus et ampli,
Si patriæ volumus, si nobis vivere cari.

Although HIPPOCRATES has no where given a complete description of the symptoms of cholera, he has made mention of it briefly and incidentally in several parts of his works: in the seventh book of his *Epidemics*, he states that choleric affections, like intermittent fevers, occur most frequently in the summer season, being superinduced by improper articles of food, such as pork imperfectly boiled, pulse, potherbs, summer fruits (especially pompions), excessive indulgence in old fragrant wine, shell fish (such as the zepia, and the like.) It appears, therefore, that indigestion and repletion, with crudities, are the causes first suspected of occasioning this disease. In the fifth book he relates an interesting case of cholera: a native of Athens, he says, was seized with cholera, and had painful discharges upwards and downwards, neither of which could be stopped; his voice failed; he could not be raised from his bed; his eyes became misty and hollow; and along with these symptoms there were spasms and singultus. He took hellebore in the decoction of lentils, and afterwards drank again as much as he could of the decoction of lentils: he then vomited, and became cold, when he was immersed in hot water up to the pudenda, until the upper extremities regained their heat, and he recovered. Next day he took a draught consisting of polenta (toasted barley meal) sprinkled upon water. Although Hippocrates has merely related the circumstances of the case as they happened, without stating whether or not he approved of the administration of the hellebore, he is severely censured by Cælius Aurelianus for expressing himself in such equivocal terms with regard to a practice which the latter pronounces to be most dangerous, and in fact wholly inadmissible. Upon this point, the bolder practitioners of the present day, who freely administer their darling cholagogue, calomel, in such cases, probably will not think the practice here mentioned deserving of the strictures which Cælius bestows upon it. Although seemingly sanctioned by such high authority, it does not appear, however, to have met with any imitators in ancient times.

Cornelius Celsus begins his account of cholera with stating that it is a disease common to the stomach and intestines, being characterised by vomiting and dejections, with inflation and tormina of the bowels, and the discharge upwards and downwards of bile, at first like water, then like water in which recent flesh had been washed, sometimes white, or black, or of various colours. In addition to these symptoms, there are contractions of the legs and hands, urgent thirst, and syncope; so that it is not to be wondered at if sudden death be often the consequence of it: and yet, he adds, there is no disease which is relieved by simpler means. Wherefore, as soon as these symptoms appear, the patient is to be made to drink a large draught of tepid water, and vomit. The water, he remarks, will generally have this effect: but even if it does not operate in this way, it will still prove beneficial by diluting the corrupted matters in the bowels, and part of the cure will be accomplished by the suppression of the vomiting; yet if the water produces vomiting, the patient must abstain from all other drink. If tormina be present, the region of the stomach is to be fomented with cold and liquid fomentations; but if they are seated in the belly, tepid or moderately hot ones are to be applied to it. When the discharges from the stomach and bowels, and the thirst, are urgent, but the matters brought up are still somewhat unconcocted, the time is not yet arrived, he says, for administering wine; but water, not cold, but rather tepid, is to be given: and pennyroyal out of vinegar is to be applied to the nostrils, or polenta sprinkled on wine, or mint not yet ripe: but when the crudities are all evacuated, we have reason to apprehend *deliquium animi*, and it will then be proper to fly to the use of wine. It must be light, fragrant, and diluted with much cold water, and is to be given with polenta or honey; and as often as the discharges from the stomach and bowels take place, some wine and water is to be given to restore the strength. Erasistratus, he says, directs to give at first a draught containing three or five drops of wine, but afterwards, by degrees, undiluted wine. Upon this practice he remarks, that if the wine was given from the commencement of the attack, he was right in beginning with a few drops, while there was reason to be apprehensive of the crudities; but if he fancied that great debility was to be removed by so insignificant a dose, he was entirely mistaken. When the patient is fairly emptied, and there are contractions of the limbs, he recommends to interpose a draught of wormwood. When the extremities are cold, he directs to anoint them with hot oil, to which some wax has been added, or to apply warm fomentations to them; and if the disorder is not thereby removed, to apply a cupping instrument or mustard over the stomach. When the violent symptoms have subsided, the patient is to be allowed to sleep: he must abstain from drink next day; take the bath on the third; and get gradually restored by proper food. When fever comes on after the suppression of the cholera, he recommends to open the bowels, and afterwards give food and wine. He concludes with remarking, that the disease is an acute one, in which the intestines and stomach are principally concerned, but in which it is not always easy to say what particular part is most affected.—Lib. iv. c. 11.

Aretæus defines cholera to be a violent retrograde flow of the matters in the whole body upon the gullet, stomach, and bowels, being a most acute disorder. It is attended, he says, with discharges upwards and downwards: upwards, of the matters collected in the stomach, and downwards, of the fluids in the bowels; these consisting of liquid fæces, having a very offensive smell, for protracted indigestion is the precursor of the disease; and, when these are evacuated, the discharges consist first of phlegm, and then of bile. At first the evacuations are not attended with much pain, but afterwards there is straining of the stomach and griping of the bowels; and, if the disorder get worse, the griping becomes more severe, there is deliquium, loss of strength, inquietude, and anorexia; or if any food be taken, it brings on nausea and vomiting of yellow bile, and the alvine discharges partake of the same character; there are spasms and contractions of the muscles in the legs and arms; the fingers are bent; there is vertigo and hiccough; the nails are livid; there is general frigidity, coldness of the extremities, and rigors. But when the evil approaches its termination, the patient is in a profuse sweat; black bile is discharged upwards and downwards; the urine is retained from spasms of the bladder, or rather no urine is secreted, all the fluids being determined to the bowels; there is loss of speech; pulse very small and very rapid, as in syncope; continued, but unavailing, strainings to vomit; and frequently tenesmus; concluding with a most painful and miserable death, brought on by the spasms, suffocation, and retching. The disease, he adds, is most common in summer; next in autumn, less so in spring, and least of all in winter. Young men and adults are most subject to it; old men least of all; children more so, but it does not prove mortal to them.—*De Morb. Acut.* ii. 5. With regard to the treatment, he pronounces it unsafe to stop the discharges, because they consist of unconcocted matters; and lays it down as the proper indication of cure to facilitate the evacuations, or to promote them, by giving tepid water to drink, frequently, but in small quantities: when there are gripes and coldness of the extremities, he directs to rub the belly with calefacient oils, to dispel flatulence, and the limbs, as far up as the knees, with the same, to restore the heat. Such is the mode of treatment when fæces are evacuated downwards, and bile upwards; but when all the remains of the food have been passed, and when the discharges upwards and downwards consist of bile, attended with straining, anxiety, inquietude, and loss of strength, he directs to give two or three small cupfuls of cold water, to act as an astringent on the belly, stop the discharge, and cool the burning heat of the stomach. When what is drunk is vomited up, this is to be frequently repeated; for the cold water soon gets warm in the stomach, and is then vomited up; but there is a constant desire for cold drink. But if the pulse sink to the lowest state, and become very rapid; if the forehead, neck, and all parts of the body, overflow with sweats; if the discharges from the belly continue, and the stomach vomit up every thing, with strainings and *deliquium animi,* he directs to add to the cold water a small quantity of a fragrant and astringent wine, in order to refresh the senses, and impart strength and nourishment to the body, and stop the evacuations; some fresh polenta may be sprinkled upon it. But if the sweats and contractions, not only of the stomach, but also of the tendons, become more urgent, and attended with hiccough; if the feet be drawn in, the discharges from the bowels become more frequent, the patient's countenance turn dark and clouded, and the pulse become insensible, there is then no time for procrastination, but a copious draught of cold wine is to be given: not, indeed, of undiluted wine, lest it bring on intoxication, and prove injurious to the nerves, but along with other food; and pieces of bread soaked in it are to be given; and we must also administer the usual restoratives in cases of syncope, such as astringent fruits, namely, services, medlars, quinces, and dried grapes. But if all these things are vomited up, and nothing remain on the stomach, we must return to hot food and drink; for sometimes the change will stop the evil, but what is given must be very hot. When none of these things will stop the discharges, he directs to apply a cupping instrument, with much heat, to different parts of the back and belly, yet so as not to occasion pain or raise blisters: sometimes, he adds, the motion of gentle gestation will rouse the patient, restore animation, and make the food remain in the stomach. But if all the symptoms are exacerbated, he recommends to try astringent applications to the chest and belly; such as dates soaked in wine, acacia, and hypocistis, which are to be mixed with rose cerate, spread upon a linen cloth, and laid upon the abdomen. He also recommends various stimulant applications to the chest, feet, and legs, to restore heat when these parts are cold. If by these means the sweats, vomitings, and purgings be stopped, if the pulse improve, and the heat be restored, sleep will concoct what remains of the disorder, and the patient is to be bathed on the second or third day, and return to his customary mode of living: but if, on the contrary, the vomiting continue, the sweats flow incessantly, the patient become cold and livid, the pulse sunk and stop, it will be well for the physician to make good his retreat.—

*Curat. Morb. Acut.* ii. 5. It may be proper to mention that Aretæus belonged to the pneumatic sect.

We now proceed to CÆLIUS AURELIANUS, whose account of the disease is highly interesting and important, as containing a full exposition of the practice approved of by the sect called the *methodical*, with free animadversions and strictures on the plans of treatment recommended by the other authorities. I have to regret, however, that my limits oblige me to give merely an abridgment of his opinions. ASCLEPIADES, he says, defined cholera to be "a rapid and acute discharge of fluids, occasioned by the concourse or obtrusion of corpuscles in the stomach and bowels, and generally brought on by indigestion." To this definition he states certain objections, and prefers the one given by SORANUS, the founder of the methodical sect: "The choleric passion is a resolution of the gullet and stomach, with imminent danger of the intestines." The precursory causes of it, he says, are intoxication, drinking of improper medicines, or waters of a heating nature: the agitation on board of a ship at sea; but more especially protracted indigestion, brought on by unusual and high-seasoned food. He gives a very minute description of the symptoms: it is commonly preceded by weight and tension of the stomach, anxiety, restlessness, want of sleep, pain and borborygmi in the intestines; then there is pain in the stomach, discharges from the bowels affording no relief; fœtid eructations, nausea, salivation, a weight about the chest, and loss of strength in the limbs; and, when the disorder is fairly set in, incessant vomiting, first of corrupted food, phlegm, and yellow bile, then of bile resembling the yolks of eggs, afterwards of a deep green colour, like leeks or verdigris, and at last black; great disorder and pain of the bowels, and discharges from them like the matters vomited up, namely, frothy and very acrid. As the disease proceeds, the discharges from the bowels become watery, and sometimes like the washings of flesh; but with these there are generally whitish and nutritious matters; the pulse becomes rapid, the limbs cold, and the countenance livid; there is great heat and unquenchable thirst; the respiration very hurried; there are contractions of the limbs, with spasms of the tendons, legs, and arms; the præcordia are retracted upwards, with pain resembling that of ileus; sometimes the alvine evacuations are bloody; the countenance is emaciated and thin, the eyes red, and at last there is singultus. He adds, that it had been called an acute and most rapid disorder, so as seldom to be prolonged to the second day. The gullet, stomach, and bowels, are the parts most affected; but all the rest of the body suffers sympathetically. He directs, in the first place, to administer a draught of tepid water, in order that the corrupted matters, resembling a poison, may be cleared away by vomiting: but if these are all evacuated, the patient is to be kept in the same posture, as motion excites the discharge of the fluids; he then recommends frictions and ligatures to the extremities, and the application, over the pit of the stomach, of sponges squeezed out of cold water, and afterwards out of sour wine, diluted; and the face is to be wiped with the same, in order to resuscitate the patient. With the same intention, we are to use odoriferous things, fan him, and apply cataplasms of a cooling nature along the chest and belly; and, in order that the patient may not be disturbed by changing them frequently, he directs to use sponges squeezed out of cold water, which are to be cooled when they become warm: but, if the pains are excruciating, he recommends, instead of the cold sponges, to use some anodyne, and then the parts are to be covered with clean wool, or sweet oil and hot water are to be poured upon them. In like manner, to relieve the contractions, the limbs are to be covered with wool, or hot cloths are to be wrapped about them. Dry cupping is also to be performed, in succession, on different parts of the belly and back; cold water is likewise to be given to drink at intervals. But if the prostration of strength and resolution gain ground, some food must be immediately given; such as bread washed in cold water, halica, or rice out of water, or sour wine, or soft eggs previously infused in vinegar, and a dry pap. If these things are rejected, they must be repeated at intervals, three or four times; and cupping instruments, having large mouths, are to be applied, with much heat, over the orifice of the stomach. He enjoins to observe a proper medium in the administration of all things, as it frequently happens that the patient is oppressed by having too much given to him. When the vomiting has been stopped for some time, and the discharges from the bowels have become less frequent, he recommends to give things to rouse the appetite; such as pears, quinces, damascenes, the breasts of birds not fat, and pickled olives. When the patient's strength is so far recruited, he permits to give him bread out of austere wine diluted with water, or wine itself, but in spare quantity, along with other food. Finally, when fever supervenes, he directs, if the patient's strength permit, to abstain from food for one day, or, at all events, to give it only during the times of remission: the diet is to be well regulated for some time, and, as soon as the patient recovers his strength, the bath is to be resorted to. He then passes in review the methods of practice recommended by Hippocrates, Diocles, Praxagoras, Erasistratus, Asclepiades, and Heraclides Terentianus; but we can only afford room for a few of his strictures on them.

He blames Diocles for restricting the administration of hot water to the winter season; whereas, he affirms that it is beneficial at all seasons, by relaxing the stomach and occasioning vomiting. Although Erasistratus properly approved of tepid water for exciting vomiting and diluting the bile, Cælius condemns his practice of using the bath, and fomentations along with it: it appears from him that the use of the bath had found abettors likewise in Praxagoras and Asclepiades. The latter he blames for administering wine too early. He speaks with disapprobation of the empirical practice of Serapion and Heraclides, who gave pills of hyoscyamus, anise, and opium, for stopping the discharges. It is worthy of remark in this place, that the famous Roman empiric, Marcellus, who flourished about the middle of the third century, recommends pills containing opium, myrrh, henbane, &c. for the cure of cholera.—*De Medicam.* c. xxx. We have mentioned above, that he blamed Hippocrates for sanctioning the administration of hellebore.—*De Morb. Acut.* c. xix.

With Cælius Aurelianus I may join Octavius Horatianus, although of a later age, as he appears to have belonged to the same medical sect. It is proper to know that this author is called Theodorus Priscianus in the Aldine edition of his works. He says of cholera, that it is "omnibus acutis ægretudinibus velocior;" for that he had often known it prove fatal within the space of one day, by bringing on sudden vomitings and discharges from the bowels, with great pain of the præcordia or whole body, so that all the inward substance is poured forth. As soon, then, as the noxious humours get concentrated about the præcordia, occasioning intolerable pains and a sense of suffocation, he advises to give forthwith tepid water to drink, and procure vomiting thereby: but when the rush of fluid by both passages becomes dreadful, he directs to avert the danger in the following manner: hot oil is to be poured over the whole body; ligatures are to be applied to the limbs; the tendons and parts undergoing contractions are to be wrapped in wool squeezed out of hot oils; or a cerate, or calefacient oils, such as those of spikenard, privet, the gleucinum, and diluted oil of iris, may be used for this purpose. When the strength is gone, and the case seems almost desperate, he recommends to attempt resuscitation by fragrant odours, and by giving suitable food, such as the juice of lentils with vinegar, soft eggs, dried grapes, crumbs soaked in wine, &c. He adds, in strong terms, that in such a case he would give cold drink, snow water, young pigeons, and partridges: he likewise speaks favorably of the cataplasms for stopping sweats. He remarks that, when fever supervenes, it is often carried off by a critical discharge of blood.—*Ad Euporist,* lib. ii. c. 13.

We have to regret that GALEN, so remarkable for extent of information, acuteness of judgment, and love of truth, has no where explained his opinions respecting the nature of cholera so fully as could have been wished: we are, therefore, reduced to the necessity of gathering his sentiments upon this subject from his occasional allusions to it in different parts of his numerous writings. He relates, with seeming approbation, that ASCLEPIADES directed to administer tepid water freely at the commencement, until all the corrupted matters be cleared away. When the strength is fast sinking, it appears that he approved of giving wine and musk, or wine alone diluted with cold water, or a piece of bread soaked in wine; he also recommends, in such cases, to apply a cupping instrument to the scrobiculus cordis. When the patient's strength is so far recovered, he directs to use the cold bath; and afterwards to give astringent food, old wine, and asses' milk. See *de Med. sec. loc.* lib. viii., *de Rat. Vict., Comment. in Hippoc. de Vict. Acut.*, &c.

Of the Greek authorities on medicine subsequent to Galen, the one most distinguished for originality of thought and observation is ALEXANDER of Tralles, whose account of cholera is therefore particularly deserving of our attention. In the first place, then, with regard to the etymology of the term, he differs from most other writers, in not deriving it from the Greek word signifying bile (χολὴ), but from the name of the intestines (χολαδες), because they are the principal seat of this disorder. He calls it a most acute disease, apt to superinduce syncope and a great collapse of the vital powers, and therefore demanding the most prompt and immediate remedies. The disease, he says, may arise from more causes than one; such as too much food, which has not been properly digested; such articles of food and drink as prove injurious to the stomach, as pompions, fatty, sweet, and oily things; a redundance of bile in the system; and the unseasonable application of cold, or the taking of cold drink. He describes the treatment of all these cases fully, according to the nature of the procatarctic cause.

1st. With regard to the treatment of cholera arising from an excess of food which has not been properly digested.

When the patient has not great evacuations, either from the stomach or bowels, but suffers most from nausea and lancinating pains, he directs to promote the discharges upwards and downwards, by administering honied water, or, if it is not at hand, large draughts of tepid water. In order to increase the disposition to vomit, he recommends to dip the fingers, or the feathers of a goose, in oil and water, and tickle the throat with them. When all the crudities are brought off, he directs to apply soothing or healing embrocations to the hypochondria, such as hot sweet oil and oil of spikenard, and then to allow the patient to sleep. When, after enjoying rest, the fever is found to be gone, he recommends to put the patient into a bath *quam primum*, and give some food; taking care, however, to avoid indigestion.

2d. The second species, which arises from disorder and atony of the stomach, and is attended with immoderate discharges both from the stomach and bowels, is to be remedied by a regimen and medicines calculated to strengthen and resuscitate the powers of the stomach. With regard to the diet, then, a piece of bread soaked in a fragrant wine is to be given, especially if the patient be free from fever, and appear to be cold and debilitated; and still more so if the disorder be brought on by eating pompions. When the cause which infests the stomach is not of a cold nature, but fatty, sweet, or oily; if attended with fœtid eructations, and if the patient suffer from fever, and is in the prime of life, he forbids to give the bread soaked in wine, but in such preparations as oxycrate, hydromel, and the wine prepared from unripe grapes: but if the vomiting is prolonged, he particularly recommends to give a decoction of mint, which he praises as being an efficacious stomachic, and well adapted for this affection. When, however, the powers of the system sink, the extremities become cold, and there are spasms and contractions, it will be proper, he says, to add some wine to the decoction; for, of all things, wine is most calculated to restore the powers when sunk; and he adds, that he had known many cases in which the patient, after being despaired of, had escaped the danger by drinking wine alone. For the external applications he gives minute directions, which I need not enter upon, as most of his preparations are such as we are now little familiar with, and for which the ingenious practitioner will readily find substitutes, when he understands the principles upon which they were applied: suffice it, then, to say that, when the extremities are cold, he directs to rub them with calefacient oils, and to lay to the pit of the stomach applications of an astringent and tonic nature. He also speaks highly of dry cupping with much heat, or even of cupping with scarifications, when there appears to be any chronic inflammation or hardness about the hypochondriac region.

3d. Cholera from a redundance of bile is recognised by the discharges upwards and downwards partaking of this character, and by there being gnawing pains and heat about the præcordia, with strong thirst and roughness of the tongue. It is most apt to occur in the summer season, especially in those who live upon a heating regimen. In this case, he says, the great indications of cure are to moisten and cool, and, therefore, the food, drink, and applications should be calculated to fulfil these purposes: he directs, then, to give strained ptisan properly cooled, and also cold lettuce with some vinegar, and in like manner savoury, bread out of water, certain of the testacea, hard fish, and the like. He likewise recommends astringent fruits, such as apples, pears, pomegranates, and hung grapes; also chesnuts and such like things. For drink he particularly commends cold water, given not in large quantities, but along with other food. When the strength is impaired, he approves of adding to it some vinous preparation possessed of acidity, or the Sabine or Cnidian wines. When the patient, owing to the dryness of the system, is troubled with insomnolency, he directs to administer certain preparations of opium in his drinks; he further recommends external applications to the hypochondria of a cooling, tonic, and repellent nature, such as houseleek and lettuce, prepared with crumbs of bread and rose oil, or such like. These things, he says, will stop the discharges from the bowels, change the intemperament, and rouse the appetite. In this manner, he adds, you may cure those affected with continued vomitings and immoderate discharges from the bowels, which bring on spasms and syncope, so as to endanger the life of the patient.

4th. Lastly, he says, when the bile is thrown into agitation, and occasions irritation, but no discharges worth mentioning take place, either by vomiting or from the belly, it will not be improper in this case, particularly if the patient be young and accustomed to be purged, to administer a purgative medicine, calculated to clear away the bilious humour: for this purpose he recommends scammony, along with mint, hydromel, or the like: by these means, he adds, the nausea will stop, and the food will be properly digested and distributed. And here I must digress to remark, that this rare species of cholera is described by Hippocrates as the dry cholera, and its nature is briefly explained by his commentator Galen, *de Victu Acut.* c. lxi. It is noticed by Sydenham in the following terms: "There is also a dry cholera from a windy spirit breaking out above and below, without vomiting or looseness, which I never saw but once, and that was in the beginning of this autumn (1669)." It is also distinctly mentioned by Broen, in his Animadversiones on the *Praxis Medica* of Henricus Regius, lib. iii. c. 25. Some cases of it have been related of late by our English physicians in India: Alexander concludes his account of cholera with remarking, that soft and gentle friction is particularly applicable for it, and also ligatures to the extremities. When the feet are cold or affected with spasms, he recommends to put them into hot water, or to pour hot water upon them, until they become warm and ruddy; after which they are to be wrapped in wool, to prevent them from catching cold. These things are more essentially proper when the discharges are upwards. The ligatures, he states, act upon the principle of revulsion, and are to be applied to the upper extremities when the discharges are downwards, and to the lower when they are upwards. In this manner, he says, you may cure cholera, being a most acute disease, and occasioning sudden danger. —Lib. vii. 16.

Oribasius, Ætius, and Paulus Ægineta, treat of the disease upon nearly the same principles; and as the last-mentioned author has delivered the most accurate exposition of them, we shall give a translation of the whole of his chapter on cholera.

"Cholera is an immoderate disorder of the belly, with discharges upwards and downwards, arising from continued indigestion of food. Persons are affected with indigestion, sometimes from the badness of their food, and sometimes owing to a redundance of depraved humors: wherefore, if the patient be seized with nausea and lancinating pains while the food remains undigested in the stomach, we must order him to drink tepid water, and to produce vomiting by putting a finger or a feather down the throat. We must also promote the discharge from the bowels. After the evacuation of the superfluities, he must have recourse to rest, and the application of warmth to the abdomen, by means of sweet oil with some wine, or by the oil of mastich, or of spikenard, if it is winter; and he must be allowed to sleep. After digestion, let him partake of baths and wholesome food. When the discharge is excessive, it will be sufficient, in the beginning, to try those things which are mentioned in the second Book for disorder of the stomach; but when the discharge continues, the belly collapses, and the pulse gets small and dense, we must apply a cataplasm of dates, with cold wine, acacia, the juice of hypocistis, and the pomegranate rind. For drink, give a cupful of moderately cold water to swallow, or, what is still better, a decoction of roses, wild vine, sumach, or vine shoots; or we may also give the juice of an acid-sweet pomegranate, sprinkling a small quantity of mint upon it. When the excretions are more acrid, and heat and thirst come on, we must give the seed of cucumber, with three cupfuls of water; and succory and lettuces, boiled in oxycrate, are to be used for food. When the discharge cannot be stopped, we must administer some of the austere wines: if the patient be free from fever, the Palmatian, the Arminian, or the Spathites, or that from unripe grapes, with honey; but when he has fever, the pomegranate wine, or myrtle, or celyratic hydromel, for drink; and, along with crumbs of bread, halica, or chondrus. If he loathes food from grain, he may chew some fruit, such as medlars, pears, quinces, or pomegranates; but he must spit out the fleshy and hard parts. When he cannot retain his food, let the region of the stomach be dry-cupped with a large instrument, and, while it is on, let some of the afore-mentioned food be taken. For the spasmodic contractions, apply to the muscles hot rags dipped in oil, and liquid cerates containing some castor or Sicyonian oil; but, before applying them, let the legs be bathed with hot water. If he cannot sleep, we must resort to the remedies recommended for watchfulness; and, if his strength permit, he may take a potion from poppy-heads. When the disease is on the decline, he may take the bath, and get recruited with chickens, pigeons, the feet of swine, or the like."—Lib. iii. c. 39.

I need not dwell upon the practice of the other Greek writers on medicine, as they supply no additional information. NONNUS gives a very brief epitome of the opinions of Paulus. —*Epitome*, c. 164. The last of them, ACTUARIUS, appears also to have conducted the treatment upon nearly the same principles as Paulus.—*Meth. Med.* iv. 5.

Among the Arabian authors, those most worthy of being consulted on the subject of cholera are RHASES, HALY ABBAS, and AVICENNA, who appear to write from a familiar acquaintance with the disease, although their practice is little different from that of the Greeks. I shall first explain the principles of treatment as laid down by Rhases, and at the same time introduce some remarks of a learned commentator on his works, LEONARDUS JACCHINUS, as they serve to throw some light upon the rationale of certain points of the ancient practice. When, therefore, the patient is seized with pungent and corroding pains, nausea, and tendency to vomiting, and looseness of the bowels, he directs to give copious draughts of hot water to encourage vomiting, and to administer the same in clysters. This commentator explains that the water must be hot, because cold would act as an astringent on the bowels; whereas it is required to dilute and wash away the offending matters. When the vomiting continues, Rhases advises to give warm water again, until all the bilious humours be removed, and the stomach can retain the food. As soon as the disease subsides, the patient is to be put into a bath of common water, and allowed to remain in it for the space of an hour, or as long as his strength will permit; after which he is to get food of easy digestion and subastringent wine, while the præcordia are rubbed with suitable oils. Jacchinus properly admonishes the reader, that the bath, meaning the cold bath, is utterly inadmissible while the discharges from the stomach and bowels continue. This is the treatment of a mild attack; but, when more alarming symptoms come on, he recommends to give troches of frankincense cooled in snow; to apply ligatures to the extremities, or, according to circumstances, to pour upon the limbs water congealed in snow, or to put them into hot water, and allow them to remain in it for some time; while to the region of the stomach applications are to be made, composed from sanders, camphor, roses, &c., or a linen cloth soaked in rose water congealed in snow, or an astringent cataplasm from dates, is to be applied. Old wine, diluted with the decoction of pomegranates, is to be given repeatedly until the vomiting stop. In case of syncope coming on, musk is to be given in the wine; and if the patient cannot open his mouth to swallow, it is to be poured down his throat, while attempts are to be made to resuscitate him with fragrant things applied to his nose. These and such like means are to be persevered in until the vomiting stop, when the patient is to get some food, and some fragrant wine along with it. For stopping the vomiting, he also approves of dry cupping to the præcordia. His commentator explains, that ligatures applied to the extremities act by producing revulsion; and that cold applications to the region of the stomach are only admissible when there is great heat in this quarter; wine, he says, is administered for the syncope as a restorative, and is mixed with astringents to stop the discharges.—*Ad Mansor.* ix. 62. See also his *Continens*, lib. xi., and *Divisio*, c. 60.

Haly Abbas defines cholera to be an evacuation of bile by vomiting, arising either from a superfluity of indigestible food, or from the bad quality of the same. The symptoms are vomiting, discharges from the belly, pungent pains in the stomach and bowels, deliquium, contraction of the features, coldness of the extremities, and so forth.—*Theor.* ix. 25. With regard to the treatment, he inculcates it as a settled principle that the discharges, upwards and downwards, are not to be rashly stopped, provided they do not become excessive, or the strength sink; but, on the contrary, that they are to be encouraged by giving the patient warm water and the oil of sweet almonds. But if they proceed to an alarming extent, he recommends to give a syrup of mint, an infusion of sebesten plums or quinces, or a prepared syrup of pears; he also directs to apply to the region of the stomach a plaster composed of myrtles, citrons, vinegar, rose oil, camphor, and the like. But if the disorder increase to such a degree that the extremities become cold, and the patient falls into a state of syncope, he recommends to sprinkle cold rose water on the face, to apply ligatures to the extremities, to rub the feet with stimulant oils, or to apply the same to the region of the stomach. When he recovers his senses, he is to get citrons, or apples, or bread soaked in diluted wine, or the like. When the patient experiences a strong sensation of heat in the stomach, he directs to lay to the scrobiculus cordis a cloth wet in rose water, or a solution of camphor congealed in snow. When the fluid evacuated is of a pituitous nature, he recommends to give a wine medicated with musk, the aromatic syrup of roses, or the trochicks of frankincense: these consist of frankincense, cubebs, cloves, camphor, &c., which are to be taken in an astringent or aromatic syrup of apples. When the discharges still continue, he recommends, in addition to the ligatures to the extremities, to rub the hands and feet strongly, and to plunge them into cold water. Dry cupping over the stomach is also said to be highly efficacious. After the disease is stopped, he enjoins the necessity of carefully regulating the diet.—*Practica,* vii. 14.

Respecting the practice of the other Arabians, it will be sufficient to mention that AVICENNA and ALSAHARAVIUS follow closely the principles of treatment laid down by Rhases, or, rather, all draw from the same fountains of knowledge. SERAPION approves of the early exhibition of tepid water, but he does not describe the other parts of the treatment minutely. In critical evacuations of yellow bile, when the strength is good, he recommends bloodletting: this, however, cannot be held to sanction the practice in cases of cholera properly so called.—Lib. iii. c. 14.

Although it is not my intention to deliver the modern history of this disease, I am induced to quote the following account of it, given by the illustrious SYDENHAM, whom I

have always looked upon as the first of our English authorities on medicine: he says, "I have found, by much consideration, and by manifold experience, that if, on the one hand, I should endeavour to expel these sharp humours that are the fuel of the disease by cathartics, I should do just as he that endeavours to quench fire with oil, seeing that the operation of the most gentle purge would but cause further disturbance, and raise new tumults; and, on the other hand, should I restrain the first effort with narcotic medicines and other astringents, whilst I hindered natural evacuations, and detained the humour against nature, the sick would undoubtedly be destroyed by an intestine war, his enemy being kept in his bowels: *for these reasons, therefore, I thought I must go in the middle way, that I might partly evacuate, and partly dilute the humour.* I found out this method several years ago, and have long experienced it, and have by it many times reduced the disease to good order. A young chicken is boiled in about three gallons of spring water, so that the liquor has scarce any relish of the chick; the sick is ordered to drink several large draughts of it, a little warm, or, for want of it, posset drink; at the same time a good quantity will serve for several clysters, to be given successively, until all the broth is consumed, and evacuated upwards and downwards; an ounce of the syrups of lettuce, violets, purslain, or waterlily, may be mixed now and then with the draughts and clysters, though the broth will do very well without such addition; so the stomach being often loaded with a considerable quantity of the liquor, and, as I may say, turned, and the injection of clysters being repeated, the sharp humours are either cast out, or, their acrimony being taken off, they are reduced to a due temper, the filth being ejected by their means, which requires three or four hours. Some anodyne medicine perfects the cure."

I need scarcely remark how well the views of this ingenious physician accord with those of the most distinguished of the ancient authorities: I mean Celsus, Aretæus, Cælius Aurelianus, Paulus Ægineta, Rhases, Haly Abbas, and Avicenna. These diligent observers of the phenomena of health and disease, viewing the preternatural evacuation of the animal fluids as an effort of nature, to relieve the system of certain deleterious matters by which it is oppressed, thought it unwise either rashly to interrupt her operation, or to hurry it on precipitately: they, therefore, disapproved alike of strong evacuants and astringents at the commencement, and recommended to be content with moderating the violence of the attack by soothing the affected parts, and obtunding the acrimony of the fluids which give rise to the irritation and disturbance. These were their general views of the principles upon which the treatment ought to be conducted; and I have good reason for knowing that, not more than twenty years ago, the most experienced English practitioners in the East Indies treated the disease in this manner; and I may add, that I myself have always found this the safest way of managing those cases of sporadic cholera which we occasionally meet with in this country. In saying this, I would beg to disclaim all pretensions to set myself up as a censor on the different practice of those who have been called upon to conduct the treatment of the disease under much more alarming aspects than I have ever witnessed; but at the same time I cannot conceal the painful impression of the uncertainty of medical experience lately produced upon my mind, upon reviewing the discordant results reported to have been derived from the most opposite remedies in this disease, after the ancient practice in such cases had fallen into neglect. One physician assures us that he had treated the disease successfully by relieving the system of its load by copious depletion: another says, that he had found this a most fatal practice, and recommends to support the strength by wine, brandy, and the most active stimulants. One directs us to arrest the discharges at once by strong narcotics, such as opium: another advises us to promote them by powerful cholagogues, such as calomel; and a third comprehends both these intentions of cure by combining the two classes of medicines together:

Quo teneam vultus mutantem Protea nodo?

We have seen that the ancient physicians, and our Sydenham, with a long train of distinguished imitators, were content with pursuing "a middle course," which certainly had the merit of being more safe, if not remarkable for boldness and decision. By simply soothing the irritation of the stomach, diluting its contents, and enabling it to expel them in an easier manner, they hoped to mitigate the violence of a disorder which they despaired of checking *in limine primo:* but when the means first tried failed to produce the intended effect, they were then prepared to have recourse to other expedients, according to the circumstances and emergencies of the case. When the ardor and disturbance about the præcordia were excessive, they used ice-cold applications, and sometimes things of a fragrant and astringent nature, to the region of the stomach. When, on the other hand, the cold was alarming, they applied hot fomentations, or sinapisms, or a cupping instrument containing a piece of burning cotton.

When the prostration of strength became very great, they all agreed in the propriety of giving wine and aromatics: but I need not go over all the parts of their practice, as I have already explained them at considerable length.

It remains to be mentioned that the ancients had no suspicion in general of cholera being contagious. The principal diseases which they admitted into this list are pestilential fevers (including smallpox and measles), elephantiasis, scabies, consumption, and ophthalmy. (See particularly GALEN, *de diff. Feb.* i. 3; ALEXANDER APHRODISIEUS, *Prob.* ii. 42; RHASES, *Ad Mansor.* iv. 24, and *Cont.* xxxiii. 5.) That cholera, as it is generally met with in this country, arising from certain procatarctic causes, is not contagious, any more than ophthalmia usually is, can admit of no doubt: but as it is well ascertained by modern as well as ancient experience, that the latter disease, under peculiar circumstances, becomes communicable from one person to another, there seems no impossibility, nay even improbability, why the other may not also be capable of assuming a similar character. It is moreover a curious fact connected with this subject, that other intestinal diseases, bearing some alliance to cholera, both in situation and symptoms, have been known to prevail extensively as epidemics, and even to become contagious. Such was the pestilential colic which prevailed in many departments of the Roman empire, during the time of Paulus Ægineta, and which is characterised by that author as being contagious; at least, Avicenna, Rabbi Moyses the Jewish translator of the Greek medical writers, and Fabius Paulinus, understand him in this sense, although his language is not quite so precise upon this point as could have been wished. Avicenna says that it was transmitted from country to country, and from person to person; and Moyses Alatinus, as quoted by Paulinus, in his learned commentary on Thucydides' description of the Plague of Athens, gives the following account of a similar epidemic which raged in Mantua during the year 1560: "Colicam, iliacamque hujus generis, *contagiosas passiones*, quas hoc in loco refert Avicenna memini me olim vidisse in civitate Mantuæ anno 1560, mensibus nimirum Augusti et Septembris, quia publice tunc temporis ejus modi passiones contagiosæ per universam civitatem quassabantur, cum sævis symptomatibus, *assiduo nempe, ac urgente vomitu bilis porraceæ in magna copia, nec non etiam æruginosæ*, lipothymia, assidua febre malignitatis non experte, et siti immensa, ac in eadem familia plurimi eo morbo oppressi inveniebantur." (*Prælectiones Marciæ*, p. 276.) This epidemic, which, it will be remarked, is here referred to as a parallel to the pestilential code described by Paulus Ægineta and Avicenna, would appear to have borne a considerable resemblance to the Indian and Russian cholera of the present day: I am, therefore, disposed to think that we ought not to be too sceptical in admitting the contagious character of the latter, although this circumstance be at variance with our general experience in regard to the disease.

---

### ITALIAN MEDICAL INSTITUTIONS AND PRACTICE.

